# Supplementary material for: Modelling Skylarks (Alauda arvensis) to Predict Impacts of Changes in Land Management and Policy: Development and Testing of an Agent-Based Model
Source: PLoS One. 2013 Jun 6;8(6):e65803. doi: 10.1371/journal.pone.0065803 (PMC3675089; doi:10.1371/journal.pone.0065803)
Supplement: Supporting Information S4 — The skylark ODdox as a zipped archive. (ZIP) [file pone.0065803.s004.zip › Skylark_ODdox/globals_enum.html]

ALMaSS Skylark ODdox: File Members


ALMaSS Skylark ODdox
 2.0


- Main Page
- Related Pages
- Classes
- Files

- File List
- File Members

- All
- Functions
- Variables
- Typedefs
- Enumerations
- Enumerator
- Macros

- CfgSecureLevel
  : configurator.h
- CfgType
  : configurator.h
- FarmToDo
  : treatment.h
- Growth\_Phases
  : plants.h
- MapErrorState
  : maperrormsg.h
- SkylarkObject
  : skylarks\_all.h
- TTypesBaitLocation
  : Rodenticide.h
- TTypesOfFarm
  : farm.h
- TTypesOfLandscapeElement
  : tole\_declaration.h
- TTypesOfPesticide
  : landscape.h
- TTypesOfSkState
  : skylarks\_all.h
- TTypesOfVegetation
  : tov\_declaration.h


- Generated on Thu Jan 10 2013 13:15:39 for ALMaSS Skylark ODdox by
   1.8.1.1
